# Supplementary material for: Zonation of the Vitis vinifera microbiome in Vino Nobile di Montepulciano PDO production area
Source: Commun Biol. 2024 Dec 9;7:1626. doi: 10.1038/s42003-024-07261-8 (PMC11628622; doi:10.1038/s42003-024-07261-8)
Supplement: Supplementary file 1 — Supplementary Information [file 42003_2024_7261_MOESM1_ESM.pdf]

Zonation of the *Vitis vinifera* microbiome in Vino Nobile di Montepulciano PDO production area

Giorgia Palladino, Enrico Nanetti, Daniel Scicchitano, Nicolò Cinti, Lucia Foresto, Alice Cozzi, Antonio Gonzalez Vara Rodriguez, Nicolò Interino, Jessica Fiori, Silvia Turroni, Marco Candela, Simone Rampelli

Supplementary Information

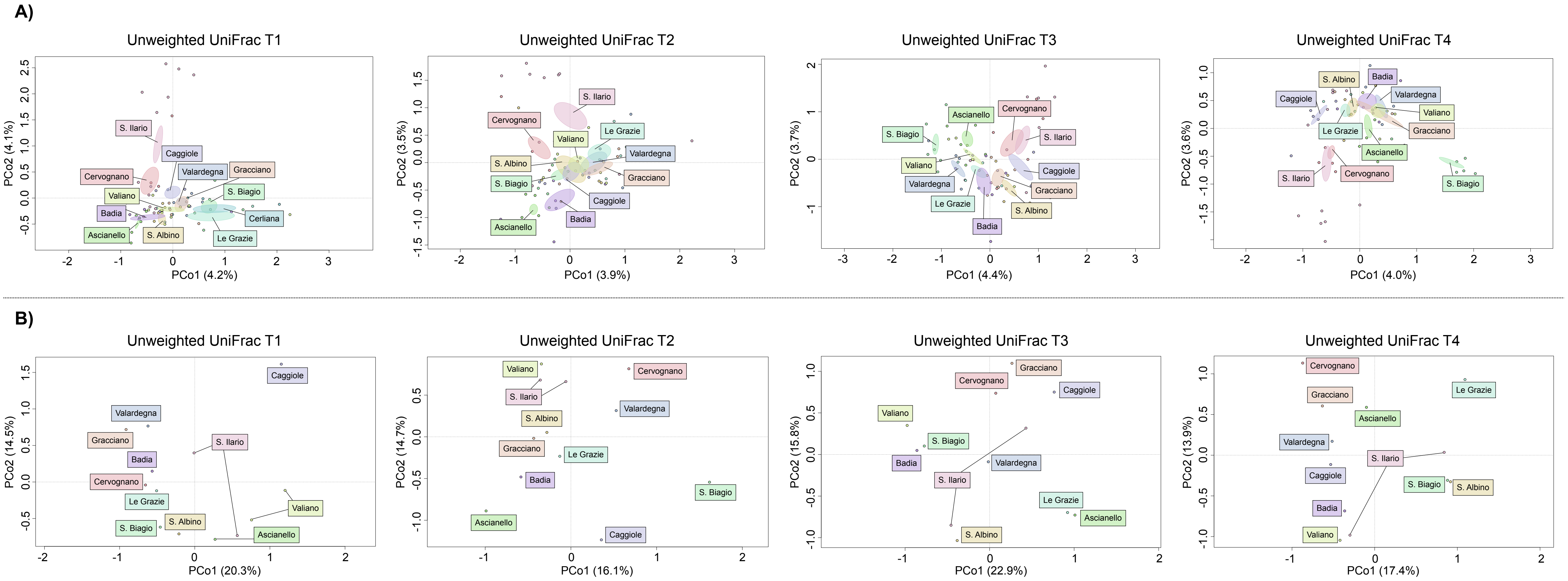

Supplementary Figure 1 - Comparison of bacterial and fungal composition of rhizospheric soil samples of Montepulciano AGUs (Additional Geographical Units) across time points (T1 to T4). (A) Principal Coordinate Analysis (PCoA) based on unweighted UniFrac distances showing the variations of *Vitis vinifera* rhizospheric soil bacterial composition in the different AGUs and time points. Procrustes test between time points, p-value = 0.005. (B) Principal Coordinate Analysis (PCoA) based on unweighted UniFrac distances showing the variations of *Vitis vinifera* rhizospheric soil fungal composition in the different AGUs and time points. Procrustes test between time points, p-value = 0.01. The first and second principal components are plotted and the percentage of variance in the dataset explained by each axis is highlighted.

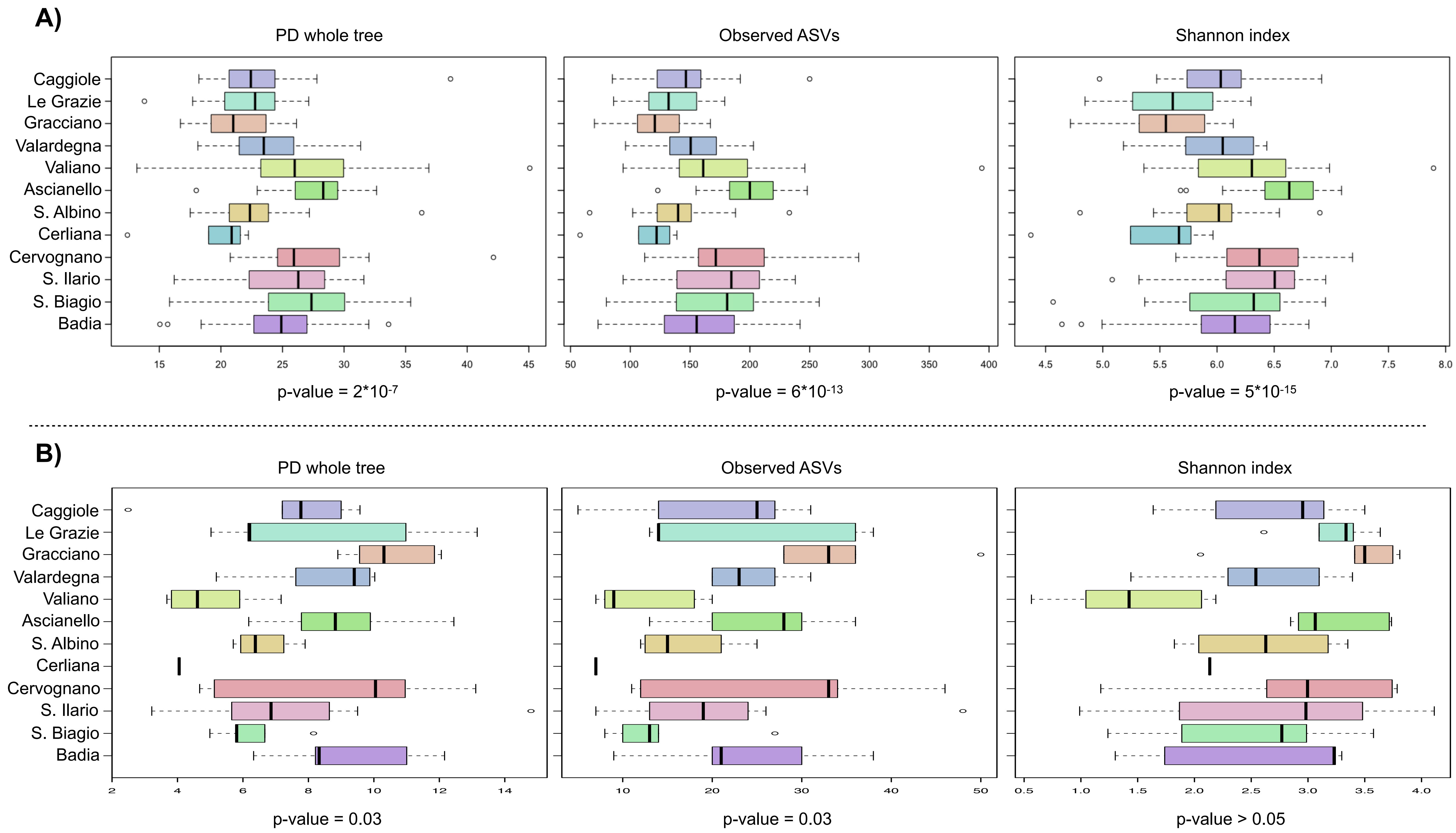

Supplementary Figure 2 - Diversity of microbial communities across Montepulciano AGUs (Additional Geographical Units). Boxplots showing the alpha-diversity distributions of the microbiome associated with samples from different AGUs for bacterial (A) and fungal (B) communities, based on the Faith's phylogenetic diversity (PD whole tree), the number of observed amplicon sequence variants (ASVs), and the Shannon index. Significant differences between AGUs are indicated below each graph (Kruskal-Wallis test).

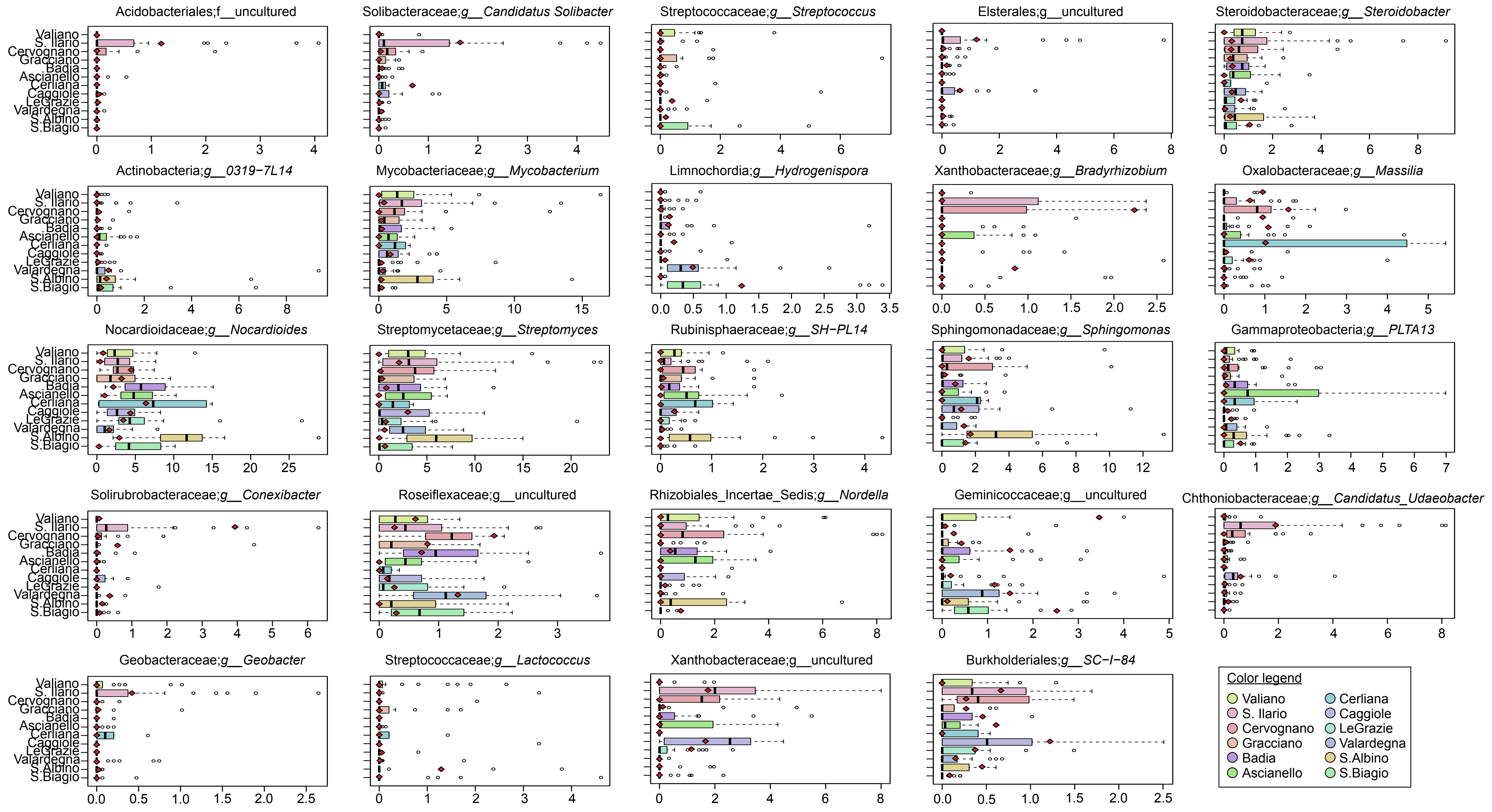

Supplementary Figure 3 - Relative abundance of the 24 bacterial taxa that discriminate among Additional Geographical Units (AGUs). The relative abundance distribution of the discriminant bacterial taxa in the rhizosphere is represented in each boxplot, whereas the mean relative abundance of the same taxa in the respective soil samples is represented with a red rhombus. Significant variations in relative abundance were assessed by Kruskal-Wallis test controlled for multiple testing using False Discovery Rate (FDR) (p-value ≤ 0.001 for all taxa). AGUs are reported in the first column of boxplots and in the color legend at the bottom right.

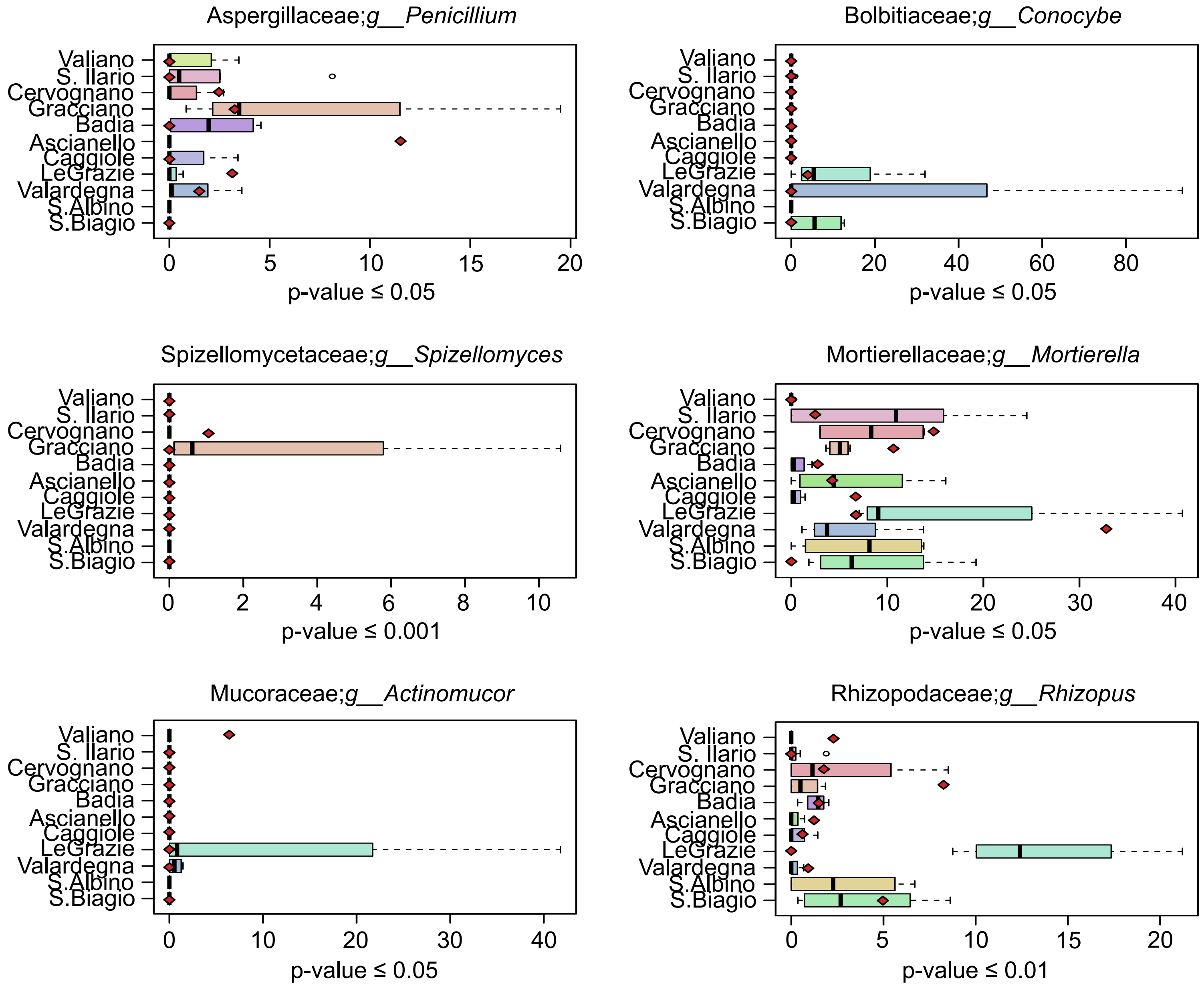

Supplementary Figure 4 - Relative abundance of the 6 fungal taxa that discriminate among Additional Geographic Units - AGUs. The relative abundance distribution of the discriminant fungal taxa in the rhizosphere is represented in each boxplot, whereas the mean relative abundance of the same taxa in the respective soil samples is represented with a red rhombus. Significant variations in relative abundance were assessed by Kruskal-Wallis test controlled for multiple testing using False Discovery Rate (FDR) (p-values are reported below each boxplot). AGUs are indicated next to each boxplot.

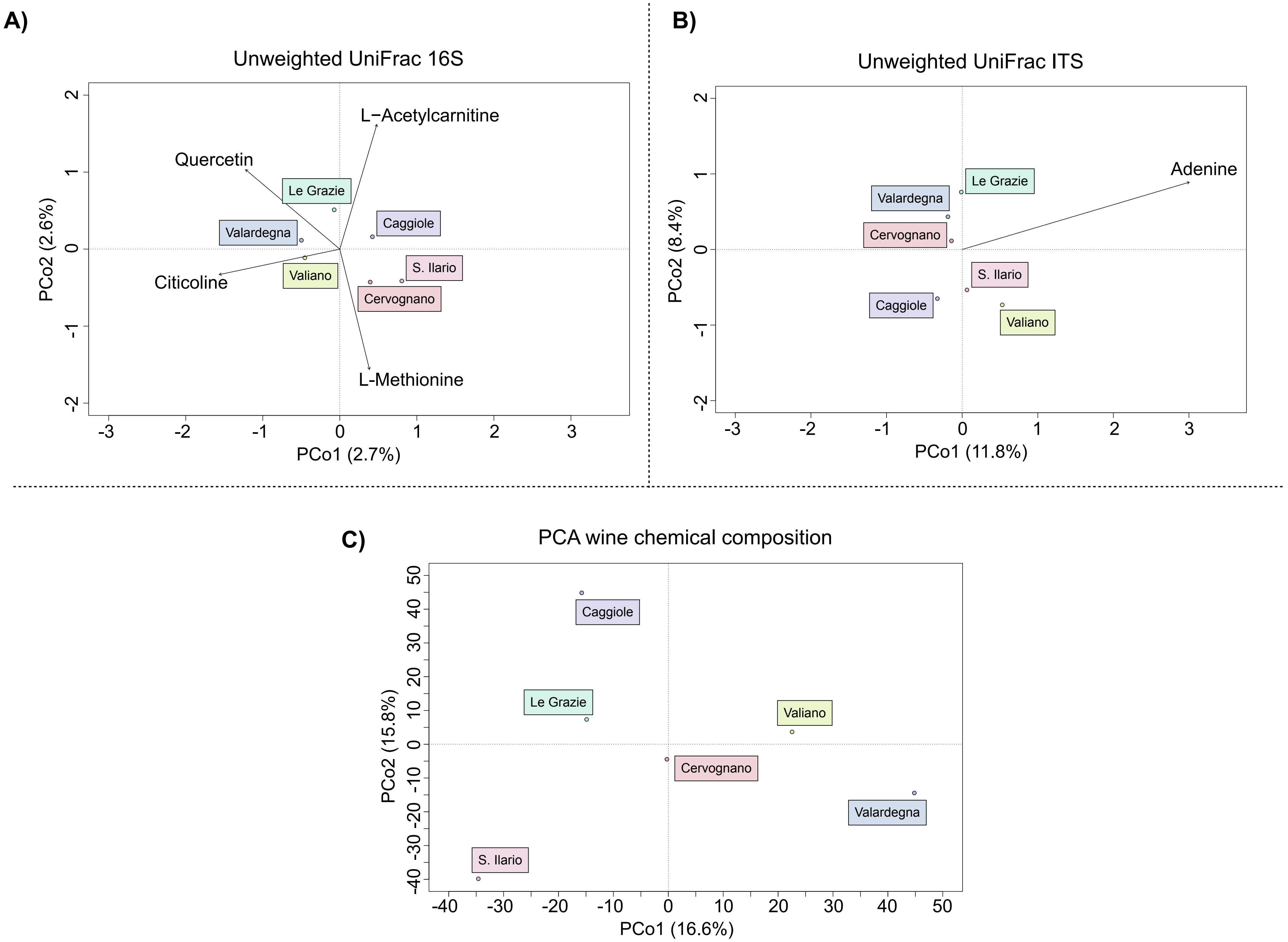

Supplementary Figure 5 - Impact of wine chemical composition on rhizospheric microbial communities of Montepulciano AGUs (Additional Geographical Units). Principal Coordinate Analysis (PCoA) based on unweighted UniFrac distances between bacterial (A) and fungal (B) profiles of *Vitis vinifera* cultivar Sangiovese rhizospheres in the different Additional Geographical Units (AGUs) of the Montepulciano territory. Only the six AGUs selected for wine chemical composition are shown. Superimposition of wine analytical components through the envfit function are shown (p-values ≤ 0.05). (C) Principal Component Analysis of wine analytical components in the six selected AGUs. In all graphs, the first and second principal components are plotted and the percentage of variance in the dataset explained by each axis is highlighted.

Supplementary Table 1 - Site and plant characteristics of all vineyards in the Montepulciano consortium. Additional Geographic Units (AGUs) are given in the first column. Plant age is given in years (y), altitude in meters above sea level (masl), and soil composition in percentage of soil type (gravel, sand, silt and clay). Soil composition was retrieved by Costantini et al. (2021). "--", no information available.

AGU	Vineyard	Plant age (y)	Rootstock	Rootstock type	Clone family	Agronomical practice	Management	Latitude	Longitude	Altitude (masl)	Soil composition (%)			
											Gravel	Sand	Silt	Clay
Valiano	Tenuta Tre Rose	>15	420 A - Gravesac	Berlanderi x Riparia	CG5 - CV20	Plow sole	Biological	43.16022892	11.93597164	337	5	47	29	24
	Palazzo Vecchio	>15	1103P, 110R, 775P	Berlanderi x Rupestris	VCR	Partial grassing	Conventional	43.1657283	11.90092418	320	5	47	29	24
Cervognano	Boscarelli	>15	1103, 110R, 420A	Berlanderi x Rupestris	CH20	Partial grassing	Integrated	43.10599529	11.85076717	301	9	42	36	22
Cerliana	La Ciarliana	>15	420A	Berlanderi x Riparia	R	Plow sole	Conventional	43.11531862	11.81196374	340	9	42	36	22
S. Albino	Fattoria del Cerro	>15	K5BB	Berlanderi x Riparia	CH20	Plow sole	Integrated	43.07135327	11.80867656	470	9	42	36	22
Valardegna	Salcheto	>15	1103P	Berlanderi x Rupestris	Prugnolo Grifo/Bravio	Partial grassing	Biological	43.08663725	11.80031461	422	9	42	36	22
S. Biagio	Le Bertille	>15	--	--	--	--	--	43.10096085	11.76677398	380	9	42	36	22
S. Ilario	Podere Casanova	>15	3309C	Riparia x Rupestris	R	Grassing	Biological	43.07415305	11.89457831	260	10	77	14	9
	Bindella	>15	110R	Berlanderi x Rupestris	VCR	Partial grassing	Integrated	43.09568433	11.86674385	310	9	42	36	22
Le Grazie	Fattoria della Talosa	>15	110R	Berlanderi x Rupestris	G76	Partial grassing	Biological	43.11157039	11.78225739	410	9	42	36	22
Ascianello	De' Ricci	>15	1103P, K5BB	Berlanderi x Rupestris, Berlanderi x Riparia	CH20	Partial grassing	Biological	43.140338	11.791788	350	9	42	36	22
Gracciano	Fattoria Svetoni	>15	--	--	--	Partial grassing	Biological	43.13461785	11.8367263	280	10	77	14	9
Caggiole	Azienda agricola Tiberini	>15	1103P, 110R, 140R, 157.11	Berlanderi x Rupestris, Berlanderi x Riparia	SG	Partial grassing	Biological	43.12371226	11.80025501	310	9	42	36	22
Badia	Avignonesi	>15	--	--	--	--	Biodynamic	43.157433	11.81136	280	9	42	36	22

Supplementary Table 2 - Chemical soil properties in the vineyards from different AGUs. Abbreviations: N = nitrogen, P = phosphorous, K = potassium, Hum = humidity, EC = electrical conductivity, Temp = temperature

AGU	N (mg/Kg)	P (mg/Kg)	K (mg/Kg)	pH	Hum (%)	EC	Temp °C
S. Ilario	6	9	18	8.10	14.23	91	24.6
Ascianello	27	37	75	6.73	20	375	21.2
Badia	12	17	37	6.72	13	171	24.1
Caggiole	6	9	19	6.63	15	97	17.5
Cerliana	1	1	3	6.72	18.4	15	24.1
Cervognano	NA	NA	NA	NA	NA	NA	NA
Gracciano	12	17	36	8.25	13.2	177	24.3
Le Grazie	10	14	30	6.42	15.7	143	17.6
S.Albino	10	14	30	7.44	15.44	149	22.1
S.Biagio	42	59	119	6.67	17.4	596	29.6
Valardegna	10	15	31	8.45	14.60	150	23.6
Valiano	NA	NA	NA	NA	NA	NA	NA

Supplementary Table 3 - Functional plant growth-promoting (PGP) profile of the rhizosphere and soil microbiome of Additional Geographic Units (AGUs). Abundance of PGP functions identified in the rhizosphere and bulk soil samples of different AGUs using a read-mapping approach. Data were normalized in copies per million ((reads count for an enzyme in a given sample/(gene length/1000))/(n° reads per sample/10^6).

AGU	Valardegna		S.Biagio		LeGrazie		Badia		Ascianello	
Vineyard	Salcheto		Bertille		Talosa		Avignonesi		DeRicci	
Ecosystem	Rhizosphere	Bulk soil	Rhizosphere	Bulk soil	Rhizosphere	Bulk soil	Rhizosphere	Bulk soil	Rhizosphere	Bulk soil
N fixation	253.33244	253.56081	247.72462	248.112129	251.914698	242.144023	246.841611	245.896123	272.236875	255.770062
P solubilization	519.693471	513.847621	517.117393	469.877291	539.80944	520.801887	541.353565	525.087623	502.753704	477.580141
Siderophore production	117.709424	102.601028	95.1561947	86.858134	83.7740471	89.4248764	106.68441	116.10761	136.425328	133.276813
IAA production	485.630976	451.86668	494.393106	376.625372	504.947659	474.766202	492.37769	494.630377	484.131827	458.895643
ACC deaminase production	23.007601	22.7278197	21.6513326	19.5976165	22.9139776	24.6880899	21.4839648	18.7107619	18.0837785	20.6455929

AGU	S.Albino		Valiano				Cerliana		Gracciano	
Vineyard	Cerro		PalazzoVecchio		TreRose		Ciarliana		Svetoni	
Ecosystem	Rhizosphere	Bulk soil	Rhizosphere	Bulk soil	Rhizosphere	Bulk soil	Rhizosphere	Bulk soil	Rhizosphere	Bulk soil
N fixation	245.161867	237.297965	261.4787	258.75021	247.017013	289.026902	267.87289	231.754579	270.074223	266.946281
P solubilization	504.167645	427.029998	462.810473	507.034069	490.230313	499.636566	525.466851	421.721006	534.439308	530.437528
Siderophore production	92.5650446	86.6199499	145.151287	104.084526	107.48817	119.575935	108.305706	93.9227997	100.63267	130.501276
IAA production	485.80206	414.209339	461.286801	448.963185	439.289388	478.49473	559.174905	414.594039	527.122714	481.003453
ACC deaminase production	20.4542001	19.1470931	20.4485896	20.7340903	13.7356376	15.1534794	19.678259	20.6158577	22.3434965	25.2639428

AGU	Caggiole		Cervognano		S. Ilario			
Vineyard	Tiberini		Boscarelli		Bindella		Casanova	
Ecosystem	Rhizosphere	Bulk soil	Rhizosphere	Bulk soil	Rhizosphere	Bulk soil	Rhizosphere	Bulk soil
N fixation	254.70967	244.997058	248.1592	248.150919	278.514379	249.270456	259.064404	224.670145
P solubilization	532.123067	502.172868	553.042669	488.304118	545.11962	468.293636	581.538473	622.797182
Siderophore production	94.4612051	119.183709	104.591244	137.015835	120.366355	96.079104	83.2131811	108.087523
IAA production	478.426958	436.466634	524.096431	462.368216	457.238688	405.289537	497.46969	461.164674
ACC deaminase production	21.1471873	21.6917165	20.8084552	25.5832629	18.9627404	17.1426964	17.9646773	27.108519

Supplementary Table 4 - Metagenome-assembled genomes (MAGs) taxonomically assigned to previous Additional Geographic Unit (AGU)-associated genera. Only MAGs with more than 50% completeness and less than 5% contamination were considered. For each MAG (column 1), the PhylophlAn assignment is reported in column 2, the respective 16S taxonomy is reported in column 3, completeness and contamination percentages are reported in column 4 and 5, and bp size is reported in column 6.

<table>
<tr><th>Genomic bin</th><th>PhyloPhlAn assignment</th><th>16S taxonomy</th><th>Completeness %</th><th>Contamination %</th><th>Size bp</th></tr>
<tr><td>bin.290</td><td>uSGB_61472:Other:k__Bacteria|p__Actinobacteria|c__CFGB38726|o__OFGB38726|f__FGB38726|g__GGB44089|s__GGB44089_SGB61472|t__SGB61472:0.247037</td><td>Actinobacteria Unclassified</td><td>54.47</td><td>2.91</td><td>3027411</td></tr>
<tr><td>bin.412</td><td>uSGB_61528:Other:k__Bacteria|p__Actinobacteria|c__CFGB35837|o__OFGB35837|f__FGB35837|g__GGB44133|s__GGB44133_SGB61528|t__SGB61528:0.250557</td><td>Actinobacteria Unclassified</td><td>53.09</td><td>3.02</td><td>1503685</td></tr>
<tr><td>bin.26</td><td>uSGB_55849:Other:k__Bacteria|p__Actinobacteria|c__CFGB10712|o__OFGB10712|f__FGB10712|g__GGB32443|s__GGB32443_SGB55849|t__SGB55849:0.265725</td><td>Actinobacteria Unclassified</td><td>76.83</td><td>3.51</td><td>2438761</td></tr>
<tr><td>bin.320</td><td>kSGB_35016:Species:k__Bacteria|p__Actinobacteria|c__Actinobacteria|o__Micrococcales|f__Microbacteriaceae|g__Agromyces|s__Agromyces_ramosus|t__SGB35016:0.152843</td><td>Agromyces</td><td>57.4</td><td>4.10</td><td>2789788</td></tr>
<tr><td>bin.340</td><td>kSGB_31955:Species:k__Bacteria|p__Proteobacteria|c__Alphaproteobacteria|o__Rhizobiales|f__Bradyrhizobiaceae|g__Bradyrhizobium|s__Bradyrhizobium_algeriense|t__SGB31955:0.26546</td><td>Bradyrhizobium</td><td>96.65</td><td>1.80</td><td>4623281</td></tr>
<tr><td>bin.202</td><td>uSGB_49283:Genus:k__Bacteria|p__Actinobacteria|c__Actinobacteria|o__Micrococcales|f__Cellulomonadaceae|g__Cellulomonas|s__Cellulomonas_SGB49283|t__SGB49283:0.312952</td><td>Cellulomonas</td><td>57.18</td><td>0.78</td><td>1649748</td></tr>
<tr><td>bin.1</td><td>uSGB_13269:Family:k__Bacteria|p__Actinobacteria|c__Thermoleophilia|o__Solirubrobacterales|f__Conexibacteraceae|g__GGB8534|s__GGB8534_SGB13269|t__SGB13269:0.283502</td><td>Conexibacter</td><td>61.91</td><td>1.76</td><td>5199929</td></tr>
<tr><td>bin.176</td><td>uSGB_13270:Family:k__Bacteria|p__Actinobacteria|c__Thermoleophilia|o__Solirubrobacterales|f__Conexibacteraceae|g__GGB8535|s__GGB8535_SGB13270|t__SGB13270:0.243761</td><td>Conexibacter</td><td>65.18</td><td>4.97</td><td>1715402</td></tr>
<tr><td>bin.348</td><td>uSGB_13270:Family:k__Bacteria|p__Actinobacteria|c__Thermoleophilia|o__Solirubrobacterales|f__Conexibacteraceae|g__GGB8535|s__GGB8535_SGB13270|t__SGB13270:0.192258</td><td>Conexibacter</td><td>67.54</td><td>1.71</td><td>2165037</td></tr>
<tr><td>bin.396</td><td>kSGB_16435:Species:k__Bacteria|p__Actinobacteria|c__Actinobacteria|o__Cryptosporangiales|f__Cryptosporangiaceae|g__Cryptosporangium|s__Cryptosporangium_aurantiacum|t__SGB16435:0.276697</td><td>Cryptosporangium</td><td>52.12</td><td>0.85</td><td>1495776</td></tr>
<tr><td>bin.144</td><td>kSGB_33795:Species:k__Bacteria|p__Actinobacteria|c__Rubrobacteria|o__Gaiellales|f__Gaiellaceae|g__Gaiella|s__Gaiella_occulta|t__SGB33795:0.256381</td><td>Gaiella</td><td>73.59</td><td>4.78</td><td>1669372</td></tr>
<tr><td>bin.83</td><td>kSGB_33795:Species:k__Bacteria|p__Actinobacteria|c__Rubrobacteria|o__Gaiellales|f__Gaiellaceae|g__Gaiella|s__Gaiella_occulta|t__SGB33795:0.339586</td><td>Gaiella</td><td>69.63</td><td>3.03</td><td>2860656</td></tr>
<tr><td>bin.228</td><td>kSGB_12835:Species:k__Bacteria|p__Proteobacteria|c__Betaproteobacteria|o__Burkholderiales|f__Oxalobacteraceae|g__Massilia|s__Massilia_yuzhufengensis|t__SGB12835:0.35328</td><td>Massilia</td><td>54.81</td><td>2.81</td><td>2796737</td></tr>
<tr><td>bin.164</td><td>kSGB_32229:Species:k__Bacteria|p__Proteobacteria|c__Alphaproteobacteria|o__Rhizobiales|f__Phyllobacteriaceae|g__Mesorhizobium|s__Mesorhizobium_sp_YM1C_6_2|t__SGB32229:0.212502</td><td>Mesorhizobium</td><td>59.82</td><td>3.92</td><td>5194556</td></tr>
<tr><td>bin.349</td><td>uSGB_61376:Genus:k__Bacteria|p__Actinobacteria|c__Actinobacteria|o__Corynebacteriales|f__Mycobacteriaceae|g__Mycobacterium|s__Mycobacterium_SGB61376|t__SGB61376:0.35328</td><td>Mycobacterium</td><td>98.54</td><td>0.97</td><td>2070293</td></tr>
<tr><td>bin.19</td><td>kSGB_853:Species:k__Archaea|p__Thaumarchaeota|c__Nitrososphaeria|o__Nitrososphaerales|f__Nitrososphaeraceae|g__Nitrososphaera|s__Candidatus_Nitrososphaera_gargensis|t__SGB853:0.320287</td><td>Archaea</td><td>64.29</td><td>2.76</td><td>2948997</td></tr>
<tr><td>bin.378</td><td>uSGB_55847:Family:k__Bacteria|p__Actinobacteria|c__Actinobacteria|o__Corynebacteriales|f__Nocardiaceae|g__GGB38223|s__GGB38223_SGB55847|t__SGB55847:0.265526</td><td>Nocardiaceae Unclassified</td><td>56.04</td><td>3.61</td><td>1668073</td></tr>
<tr><td>bin.111</td><td>kSGB_34149:Species:k__Bacteria|p__Actinobacteria|c__Actinobacteria|o__Propionibacteriales|f__Nocardioidaceae|g__Nocardioides|s__Nocardioides_iriomotensis|t__SGB34149:0.26546</td><td>Nocardioides</td><td>77.70</td><td>4.37</td><td>1248512</td></tr>
<tr><td>bin.197</td><td>kSGB_55752:Species:k__Bacteria|p__Actinobacteria|c__Actinobacteria|o__Propionibacteriales|f__Nocardioidaceae|g__Nocardioides|s__Nocardioides_bacterium|t__SGB55752:0.300994</td><td>Nocardioides</td><td>51.66</td><td>2.59</td><td>1740489</td></tr>
<tr><td>bin.92</td><td>kSGB_15965:Species:k__Bacteria|p__Actinobacteria|c__Actinobacteria|o__Propionibacteriales|f__Nocardioidaceae|g__Nocardioides|s__Nocardioides_sp_Root122|t__SGB15965:0.254359</td><td>Nocardioides</td><td>57.42</td><td>4.42</td><td>2834555</td></tr>
<tr><td>bin.278</td><td>kSGB_11537:Species:k__Bacteria|p__Proteobacteria|c__Alphaproteobacteria|o__Sphingomonadales|f__Sphingomonadaceae|g__Novosphingobium|s__Novosphingobium_sp_Rr_2_17|t__SGB11537:0.193331</td><td>Novosphingobium</td><td>57.43</td><td>4.67</td><td>2453724</td></tr>
<tr><td>bin.307</td><td>kSGB_16407:Species:k__Bacteria|p__Actinobacteria|c__Actinobacteria|o__Streptosporangiales|f__Thermomonosporaceae|g__Thermomonospora|s__Thermomonospora_curvata|t__SGB16407:0.328965</td><td>Novosphingobium/Thermomonospora</td><td>52.49</td><td>2.58</td><td>2643601</td></tr>
<tr><td>bin.321</td><td>uSGB_55819:Family:k__Bacteria|p__Cyanobacteria|c__Cyanobacteria_unclassified|o__Synechococcales|f__Prochloraceae|g__GGB41164|s__GGB41164_SGB55819|t__SGB55819:0.248764</td><td>Synechococcales Unclassified</td><td>83.49</td><td>2.91</td><td>2104713</td></tr>
<tr><td>bin.325</td><td>uSGB_61813:Other:k__Bacteria|p__Proteobacteria|c__CFGB35932|o__OFGB35932|f__FGB35932|g__GGB44394|s__GGB44394_SGB61813|t__SGB61813:0.273629</td><td>Proteobacteria Unclassified</td><td>62.1</td><td>4.73</td><td>2190116</td></tr>
<tr><td>bin.96</td><td>uSGB_61813:Other:k__Bacteria|p__Proteobacteria|c__CFGB35932|o__OFGB35932|f__FGB35932|g__GGB44394|s__GGB44394_SGB61813|t__SGB61813:0.26803</td><td>Proteobacteria Unclassified</td><td>62.22</td><td>1.03</td><td>1350259</td></tr>
<tr><td>bin.128</td><td>kSGB_67219:Species:k__Bacteria|p__Proteobacteria|c__Alphaproteobacteria|o__Rhodobacterales|f__Rhodobacteraceae|g__Rhodobacteraceae_unclassified|s__Rhodobacteraceae_bacterium|t__SGB67219:0.372583</td><td>Rhodobacter</td><td>52.21</td><td>2.04</td><td>1857802</td></tr>
<tr><td>bin.355</td><td>uSGB_52610:Genus:k__Bacteria|p__Actinobacteria|c__Actinobacteria|o__Micrococcales|f__Microbacteriaceae|g__Rhodoluna|s__Rhodoluna_SGB52610|t__SGB52610:0.300994</td><td>Microbacteriaceae</td><td>74.22</td><td>3.53</td><td>2002286</td></tr>
<tr><td>bin.121</td><td>kSGB_17577:Species:k__Bacteria|p__Actinobacteria|c__Rubrobacteria|o__Rubrobacterales|f__Rubrobacteraceae|g__Rubrobacter|s__Rubrobacter_xylanophilus|t__SGB17577:0.300994</td><td>Rubrobacter</td><td>54.39</td><td>0.50</td><td>1851211</td></tr>
<tr><td>bin.36</td><td>kSGB_17577:Species:k__Bacteria|p__Actinobacteria|c__Rubrobacteria|o__Rubrobacterales|f__Rubrobacteraceae|g__Rubrobacter|s__Rubrobacter_xylanophilus|t__SGB17577:0.258493</td><td>Rubrobacter</td><td>60.04</td><td>3.45</td><td>3175285</td></tr>
<tr><td>bin.178</td><td>kSGB_55872:Species:k__Bacteria|p__Actinobacteria|c__Thermoleophilia|o__Solirubrobacterales|f__Solirubrobacteraceae|g__Solirubrobacter|s__Solirubrobacter_sp_CPCC_204708|t__SGB55872:0.295981</td><td>Solirubrobacter</td><td>74.11</td><td>1.94</td><td>1593893</td></tr>
<tr><td>bin.119</td><td>kSGB_24610:Species:k__Bacteria|p__Proteobacteria|c__Alphaproteobacteria|o__Sphingomonadales|f__Sphingomonadaceae|g__Sphingomonas|s__Sphingomonas_sp_URHD0007|t__SGB24610:0.35328</td><td>Sphingomonas</td><td>66.50</td><td>3.23</td><td>2636012</td></tr>
<tr><td>bin.123</td><td>kSGB_10471:Species:k__Bacteria|p__Proteobacteria|c__Gammaproteobacteria|o__Nevskiales|f__Sinobacteraceae|g__Steroidobacter|s__Steroidobacter_denitrificans|t__SGB10471:0.295981</td><td>Steroidobacter</td><td>78.95</td><td>3.41</td><td>2177673</td></tr>
<tr><td>bin.42</td><td>kSGB_10471:Species:k__Bacteria|p__Proteobacteria|c__Gammaproteobacteria|o__Nevskiales|f__Sinobacteraceae|g__Steroidobacter|s__Steroidobacter_denitrificans|t__SGB10471:0.312952</td><td>Steroidobacter</td><td>74.23</td><td>4.68</td><td>3459230</td></tr>
<tr><td>bin.126</td><td>kSGB_16111:Species:k__Bacteria|p__Actinobacteria|c__Actinobacteria|o__Streptomycetales|f__Streptomycetaceae|g__Streptomyces|s__Streptomyces_sp_1114_5|t__SGB16111:0.270747</td><td>Streptomyces</td><td>68.45</td><td>3.45</td><td>3676162</td></tr>
<tr><td>bin.173</td><td>kSGB_34439:Species:k__Bacteria|p__Actinobacteria|c__Actinobacteria|o__Streptomycetales|f__Streptomycetaceae|g__Streptomyces|s__Streptomyces_sp_TAA040|t__SGB34439:0.320287</td><td>Streptomyces</td><td>63.88</td><td>4.34</td><td>2875784</td></tr>
<tr><td>bin.43</td><td>uSGB_46364:Genus:k__Bacteria|p__Actinobacteria|c__Actinobacteria|o__Streptomycetales|f__Streptomycetaceae|g__Streptomyces|s__Streptomyces_SGB46364|t__SGB46364:0.35328</td><td>Streptomyces</td><td>78.38</td><td>3.39</td><td>2183585</td></tr>
<tr><td>bin.276</td><td>kSGB_12768:Species:k__Bacteria|p__Proteobacteria|c__Betaproteobacteria|o__Burkholderiales|f__Comamonadaceae|g__Variovorax|s__Variovorax_sp_YR216|t__SGB12768:0.320287</td><td>Variovorax</td><td>66.22</td><td>4.88</td><td>2533478</td></tr>
</table>

Supplementary Table 5 - METABOLIC output of the reconstructed metagenome-assembled genomes (MAGs). Numerical values correspond to the gradient heatmap in Figure 8.

Function	bin.1	bin.111	bin.119	bin.121	bin.123	bin.126	bin.128	bin.144	bin.164	bin.173	bin.176	bin.178	bin.19	bin.197	bin.202	bin.228	bin.26	bin.276	bin.278
C-S-01:Organic carbon oxidation - CO oxidation	0	0	0	0	0	12.3	0	6.5	0	0	17.2	0	0	11.7	0.6	0	0	0	0
C-S-01:Organic carbon oxidation - amino acid utilization	0	0	0	0	2.4	10.7	0	0	0	0	14.9	0.5	3.2	10.1	0	0	15.6	0	1.9
C-S-01:Organic carbon oxidation - aromatics degradation	0	0	0	0	0	0	0	0	0	0	0	0	0	0	0	0	0	0	0
C-S-01:Organic carbon oxidation - complex carbon degradation	0	0.7	0	2.1	3.7	16.9	0	0	1.7	0	23.6	0.9	0	16	0.8	0	0	0	3
C-S-01:Organic carbon oxidation - fatty acid degradation	0	0	0	1.6	0	12.5	0	6.6	0	0	17.4	0	0	0	0	0	18.2	0	0
C-S-01:Organic carbon oxidation - formaldehyde oxidation	0	0	0	0	0	43.5	0	23.1	0	0	0	0	0	0	0	0	0	0	0
C-S-01:Organic carbon oxidation - formate oxidation	0	0	0	4.4	0	35.4	0	0	0	0	0	0	0	33.6	0	0	0	0	0
C-S-01:Organic carbon oxidation - methanol oxidation	0	0	0	0	0	0	0	0	0	0	0	0	0	0	0	0	0	0	0
C-S-03:Ethanol oxidation	0	0	0	0	0	0	0	0	0	0	0	0	0	0	0	0	0	0	0
C-S-04:Acetate oxidation	0	0	0	0	0	0	0	16.9	0	0	44.5	0	0	30.2	0	0	0	0	0
C-S-06:Fermentation	0	0	0	0	0	15.7	0	8.4	1.6	0	22	0	0	15	0	0	0	0	0
C-S-08:Methanotrophy	0	0	0	0	0	0	0	0	0	0	0	0	0	0	0	0	0	0	0
C-S-09:Hydrogen oxidation	0	0	0	0	0	0	0	0	0	0	0	0	0	0	0	0	0	0	0
N-S-04:Nitrate reduction - narGH	0	0	0	0	0	0	0	0	0	0	58.5	0	0	39.7	0	0	0	0	0
N-S-05:Nitrite reduction - nirKS	0	0	0	0	0	0	0	0	6	0	83.7	0	0	0	0	0	0	0	0
N-S-06:Nitric oxide reduction	0	0	0	0	0	0	0	0	0	0	0	0	0	0	0	0	0	0	0
N-S-08:Nitrite ammonification - nirBD	0	2.5	0	0	12.8	0	0	0	0	0	0	0	0	54.7	0	0	0	0	10.1
O-S-01:Iron reduction	0	0	0	0	0	13.8	0	7.3	0	0	19.3	0	0	13.1	0	0	0	0	0
O-S-02:Iron oxidation:	0	0	0	0	20.7	0	0	0	0	0	0	0	0	0	0	0	0	0	0
S-S-03:Sulfur oxidation - sdo	0	0	0	0	0	0	0	0	0	0	25.4	0	5.4	0	0	0	0	0	0
S-S-04:Sulfite oxidation	0	0	0	0	0	0	0	0	0	0	0	0	0	0	0	0	32.9	0	0
S-S-05:Sulfate reduction	0	0	0	0	0	0	0	0	0	0	0	0	0	0	0	0	32.9	0	0
S-S-07:Thiosulfate oxidation	0	0	0	0	0	0	0	0	0	0	0	0	0	0	0	0	0	0	0

Function	bin.290	bin.307	bin.320	bin.321	bin.325	bin.340	bin.348	bin.349	bin.355	bin.36	bin.378	bin.396	bin.412	bin.42	bin.43	bin.83	bin.92	bin.96
C-S-01:Organic carbon oxidation - CO oxidation	0	9.2	0	1.3	0	2.1	1.2	0	3.1	23.8	2.5	0	8.4	0	0	0	0	0
C-S-01:Organic carbon oxidation - amino acid utilization	5.5	8	1.3	0	2.8	1.8	1	0	2.6	0	0	0	7.3	0	9.9	0	0.4	0
C-S-01:Organic carbon oxidation - aromatics degradation	0	0	0	0	0	0	0	0	0	0	0	0	0	0	100	0	0	0
C-S-01:Organic carbon oxidation - complex carbon degradation	0	12.7	2.1	0	0	0	0	0	4.2	0	0	0	11.6	0	0	0	0	0
C-S-01:Organic carbon oxidation - fatty acid degradation	6.4	0	1.5	0	0	2.2	0	0	0	24.1	0	0	8.5	0	0	0	0	1
C-S-01:Organic carbon oxidation - formaldehyde oxidation	0	0	0	0	0	0	0	0	0	0	0	0	29.8	0	0	0	0	3.6
C-S-01:Organic carbon oxidation - formate oxidation	0	26.6	0	0	0	0	0	0	0	0	0	0	0	0	0	0	0	0
C-S-01:Organic carbon oxidation - methanol oxidation	0	100	0	0	0	0	0	0	0	0	0	0	0	0	0	0	0	0
C-S-03:Ethanol oxidation	0	0	0	0	0	0	0	0	0	0	0	0	100	0	0	0	0	0
C-S-04:Acetate oxidation	0	0	0	0	8.3	0	0	0	0	0	0	0	0	0	0	0	0	0
C-S-06:Fermentation	8.1	0	2	0	4.1	0	0	0	3.9	0	3.2	0	0	1.6	14.6	0	0	0
C-S-08:Methanotrophy	0	0	0	0	0	0	0	0	0	100	0	0	0	0	0	0	0	0
C-S-09:Hydrogen oxidation	0	0	0	0	0	0	0	0	0	100	0	0	0	0	0	0	0	0
N-S-04:Nitrate reduction - narGH	0	0	0	0	0	0	0	0	0	0	0	0	0	0	0	0	1.7	0
N-S-05:Nitrite reduction - nirKS	0	0	0	0	0	10.3	0	0	0	0	0	0	0	0	0	0	0	0
N-S-06:Nitric oxide reduction	0	0	0	0	100	0	0	0	0	0	0	0	0	0	0	0	0	0
N-S-08:Nitrite ammonification - nirBD	0	0	0	0	15.1	0	0	0	0	0	0	0	0	0	0	0	0	4.7
O-S-01:Iron reduction	0	10.4	0	0	0	0	0	0	0	26.7	0	0	9.4	0	0	0	0	0
O-S-02:Iron oxidation:	0	70.1	0	0	0	0	0	0	0	0	0	0	0	9.3	0	0	0	0
S-S-03:Sulfur oxidation - sdo	9.3	0	0	0	0	0	1.8	0	4.5	35.1	0	0	0	1.8	16.8	0	0	0
S-S-04:Sulfite oxidation	0	0	0	2.3	0	0	0	0	5.6	43.7	0	0	15.5	0	0	0	0	0
S-S-05:Sulfate reduction	0	0	0	2.3	0	0	0	0	5.6	43.7	0	0	15.5	0	0	0	0	0
S-S-07:Thiosulfate oxidation	0	0	0	0	0	100	0	0	0	0	0	0	0	0	0	0	0	0

Supplementary Table 6 - Accession numbers of plant growth-promoting (PGP) proteins from the NCBI database.

Protein names are given in the first column.

Protein ID	NCBI accession
NifB	WP_011024080
NifE	WP_014404757
NifH	WP_010870393
NifN	WP_011241560
NifV	WP_011241567
NifU	NP_461477
phoA	NP_414917
GDH	NP_388275
EntF	WP_000077784
EntS	NP_415123
FslA	WP_003037766
ipdC	WP_035671558
aro10	NP_010668
aldH	NP_001260290
AcdS	XP_037178185
