# Supplementary material for: Zonation of the Vitis vinifera microbiome in Vino Nobile di Montepulciano PDO production area
Source: Commun Biol. 2024 Dec 9;7:1626. doi: 10.1038/s42003-024-07261-8 (PMC11628622; doi:10.1038/s42003-024-07261-8)
Supplement: Supplementary file 2 — Description of Additional Supplementary Files [file 42003_2024_7261_MOESM2_ESM.pdf]

Description of Additional Supplementary Files

File name: Supplementary Data 1

Description: The source data for Figure 3 in the paper. Excel sheet “Fig_3A_data” refers to the panel A of Figure 3, “Fig_3B_data” refers to panel B of Figure 3
